# Microscopic and molecular characterization of *Hepatozoon domerguei* (Apicomplexa) and *Foleyella furcata* (Nematoda) in wild endemic reptiles from Madagascar

**DOI:** 10.1051/parasite/2014046

**Published:** 2014-09-17

**Authors:** João P. Maia, Angelica Crottini, David James Harris

**Affiliations:** 1 CIBIO Research Centre in Biodiversity and Genetic Resources, InBIO, Universidade do Porto, Campus Agrário de Vairão Rua Padre Armando Quintas, N° 7 4485-661 Vairão, Vila do Conde Portugal; 2 Departamento de Biologia, Faculdade de Ciências, Universidade do Porto Rua do Campo Alegre FC4 4169-007 Porto Portugal; 3 Institut de Biologia Evolutiva (CSIC-Universitat Pompeu Fabra) Passeig Marítim de la Barceloneta 37-49 08003 Barcelona Spain

**Keywords:** Hemogregarine, *Sarcocystis*, Apicomplexa, Nematode, Filaria, Arthropod-borne diseases

## Abstract

Madagascar is one of the world’s top twelve “megadiversity” hot spots hosting unique and threatened flora and fauna. Parasites are a major component of biodiversity but remain largely uncharacterized in wildlife. In this study we combine microscopic and molecular assessment of hemoparasites in endemic reptile species from Madagascar. We detected three distinct parasites: the apicomplexans *Hepatozoon* and *Sarcocystis*, and filarial nematodes. The prevalence and intensity of these apicomplexans were low overall, while microfilarial infections in chameleons were relatively high. We detected mixed infections of two *Hepatozoon* haplotypes in *Madagascarophis colubrinus,* and of *Hepatozoon* and microfilariae in a *Furcifer* sp. Phylogenetic analyses of *Hepatozoon* showed evidence of prey-predator transmission, with identical sequences found in the snakes *M. colubrinus* and *Ithycyphus oursi*, and their prey *Furcifer* sp. Based on previous studies regarding the life cycle of *Hepatozoon domerguei* Landau, Chabaud, Michel, and Brygoo, 1970 in these hosts and due to their morphological similarity, we propose that this *Hepatozoon* haplotype is *Hepatozoon domerguei*. Future studies, including the examination of invertebrate hosts, are needed to verify this preliminary taxonomic identification. A distinct hemogregarine haplotype was found in *Oplurus* sp., which displayed morphologically different gametocytes, some of which were apparently inside leukocytes. The *Sarcocystis* identified from *Tracheloptychus petersi* was identical to that reported in a North African snake, indicating that the same lineage is found in geographically distinct regions. By combining morphological and genetic information, *Foleyella furcata* (Linstow, 1899) filarial nematodes were identified in several *Furcifer* chameleons. This study provides insights into the distribution, diversity and host-parasite interactions of hemoparasites in wild reptile populations from Madagascar.

## Abstract (fr)

Madagascar est l’un des douze premiers points chauds de « mégadiversité » au monde et héberge une flore et une faune exceptionnelles et menacées. Les parasites sont une composante majeure de la biodiversité, mais restent largement non caractérisés dans la faune sauvage. Dans cette étude, nous combinons des évaluations microscopiques et moléculaires des hémoparasites chez des espèces de reptiles endémiques de Madagascar. Nous avons détecté trois parasites distincts : les apicomplexes *Hepatozoon* et *Sarcocystis*, et des nématodes filaires. La prévalence et l’intensité de ces apicomplexes étaient globalement faibles, tandis que les infections de microfilaires chez les caméléons étaient relativement élevées. Nous avons détecté des infections mixtes de deux haplotypes d’*Hepatozoon* chez *Madagascarophis colubrinus*, et d’*Hepatozoon* et de microfilaires chez *Furcifer* sp. Les analyses phylogénétiques d’*Hepatozoon* montraient des signes de transmission proie-prédateur avec des séquences identiques trouvés chez les serpents *M. colubrinus* et *Ithycyphus oursi*, et leurs proies *Furcifer* sp. Sur la base d’études antérieures concernant le cycle de vie de *Hepatozoon domerguei* Landau, Chabaud, Michel et Brygoo, 1970 chez ces hôtes et en raison de leur similitude morphologique, nous proposons que cet haplotype d’*Hepatozoon* est *Hepatozoon domerguei*. Les études futures, y compris l’examen des hôtes invertébrés sont nécessaires pour vérifier cette identification taxonomique préliminaire. On a trouvé chez *Oplurus* sp. un haplotype distinct d’hémogrégarine qui montre des gamétocytes morphologiquement différents, dont certains étaient apparemment à l’intérieur des leucocytes. Le *Sarcocystis* identifié chez *Tracheloptychus petersi* était identique à celui rapporté dans un serpent d’Afrique du Nord, ce qui indique que la même lignée se trouve dans des régions géographiquement distinctes. En combinant information morphologique et génétique, le nématode filaire *Foleyella furcata* (Linstow, 1899) a été identifié chez plusieurs caméléons du genre *Furcifer*. Cette étude donne un aperçu sur la répartition, la diversité et les interactions hôtes-parasites des hémoparasites dans les populations de reptiles sauvages de Madagascar.

## Introduction

Madagascar is the fourth largest island in the world, and as one of the world’s top 12 “megadiversity” hot spots [54] hosts an almost unparalleled concentration of endemic, diverse and threatened fauna and flora [54, 88]. The native reptile fauna has a high level of endemism at the species level (about 92%), and is composed of at least 25 extant independent lineages [17, 24]. Currently, more than 400 reptile species are known in Madagascar [56], and this list will increase in the future as a result of intense research activities and widespread application of integrative taxonomic approaches [24, 56], as shown by the numerous recent species descriptions [18, 25, 69]. Parasites have been increasingly recognized as a main component of biodiversity; however, their study clearly lags behind that of their hosts [53]. Documenting the diversity of parasites is important for several reasons, since they (1) co-evolve and interact with their hosts [59, 75], (2) play an important role in structuring animal communities [31, 65], and (3) are important in ecosystems and conservation [61, 72].

Hemoparasites typically have complex life cycles, requiring more than one host to complete it. The life cycle of several filarial and coccidian parasites has been described in endemic hosts from Madagascar: *Foleyella furcata* (Linstow, 1899) [44], an onchocercid described from the chameleon *Furcifer verrucosus* (Cuvier, 1829) [7], and *Hepatozoon domerguei* (Landau, Chabaud, Michel and Brygoo, 1970) [37], a hemogregarine described from the lamprophiid snake *Madagascarophis colubrinus* (Schlegel, 1837) [38]. Both are arthropod-borne parasites, and the mosquito *Culex quinquefasciatus* (*Culex pipiens fatigans*) Say, 1823 has been used experimentally as a vector [7, 38]. *Foleyella* species have a limited geographic distribution and have been found only in the lizard families Agamidae and Chameleonidae [10]. Four species compose the genus *Foleyella*, of which *F. furcata* and *Foleyella brevicauda* (Chabaud and Brygoo, 1962) [14] are generally common in Malagasy chameleons [12]. Morphological identification to the species level is possible through analysis of adult forms [10]; however, the advent of molecular tools and the use of fast-evolving genetic markers now allow the placement of parasites in a phylogenetic framework, allowing assignment to the species level more easily [40, 55]. The genus *Hepatozoon* is part of the hemogregarine group and is one of the most common hemoparasites in reptiles [73]. *Hepatozoon* can be transmitted by direct ingestion of infected invertebrate hosts by vertebrate hosts or by prey-predator transmission through infective cysts in prey that can cause infection in receptive hosts [38]. Molecular parasitological studies in mammals and reptiles corroborate the latter mode of transmission by reporting identical parasite lineages in predator and prey hosts [49, 81], thus providing new insights into parasite-host interactions. Hemogregarines from continental African reptiles have shown high genetic diversity comprising various unrelated lineages [47, 82], compared with rather limited genetic diversity from the Seychelles islands [27]. Occurrence of these hemoparasites can be easily detected through microscopy, by observing hemogregarine gamonts inside erythrocytes and leukocytes [79], by observing onchocercid microfilarial stages in blood smears stained with Giemsa [33], and through molecular screening of host samples using parasite-specific primers [47, 62]. Parasite species can be better identified by combining genetic and morphological data [1, 29, 57]. Although this practice is currently easy to apply, and despite the wide range of parasites that can be found in reptile blood samples [28], studies using this approach to assess parasite prevalence and diversity are still generally lacking. Parasite research in Madagascan amphibian and reptile hosts has mainly focused on a few groups, such as the malarial parasite *Plasmodium* [71], monogenean polystomatids [11, 66], nematodes [35, 42], or it has been focused on well-known conservation threats such as the amphibian chytrid fungus *Batrachochytrium dendrobatidis* Longcore et al., 1999 [16, 19, 86, 87], while other groups remain less studied.

The aims of this study are to: (i) provide preliminary information on the prevalence and intensity of hemogregarines and filarial nematodes in endemic reptile species from Madagascar, (ii) place these parasites in a phylogenetic framework to determine the specificity of the detected parasite lineages by comparing them with known parasite species from different hosts and geographical locations, and (iii) to detect prey-predator transmission of *Hepatozoon* lineages by analyzing predators and their prey from this region.

## Materials and methods

### Sample collection

Samples were collected from 73 reptile specimens (Table 1) from several localities in the center and south-west of Madagascar, mostly Ranomafana, Ambalavao, Isalo, Ifaty, Toliara, and Lavenombato. For each individual a small tail tip was collected for molecular identification, and when enough blood was naturally available this was used to prepare a blood smear. Tissue was preserved in 96% ethanol. Individuals were released at the site of capture. Blood smears were air-dried, fixed with methanol and stained with diluted Giemsa (1:9 of distilled water) for 55 min.

**Table 1.** Reptile samples collected in different localities of Madagascar in 2009. Prevalence and intensity estimates for hemogregarines and filarial nematodes for each reptile species.

Host group	Host species	*n*	Hemogregarines		*Foleyella furcata*	
			Prevalence	Intensity (%) ± *SD* (min − max)	Prevalence	Mean Intensity (%) ± *SD* (min − max)
Chameleon	*Calumma crypticum*	3				
	*Calumma gastrotenia*	1				
	*Calumma nasutum*	1				
	*Calumma oshaughnessyi*	3				
	*Furcifer* sp.	18	1 (6%)	0.35	7 (39%)	0.26 ± 0.24 (0.02 − 0.81)
	*Furcifer antimena*	1				
	*Furcifer lateralis*	9			1 (11%)	1.34
	*Furcifer oustaleti*	12			2 (17%)	0.31 ± 0.17 (0.14 − 0.47)
	*Furcifer verrucosus*	3			3 (100%)	0.10 ± 0.04 (0.05 − 0.14)
		51	1 (2%)		13 (25%)	
Lizard	*Blaesodactylus sakalava*	1				
	*Chalarodon madagascariensis*	1				
	*Lygodactylus pictus*	1				
	*Oplurus* sp.	1	1 (100%)	0.07		
	*Tracheloptychus petersi*	1				
		5	1 (20%)			
Snake	*Compsophis laphystius*	1				
	*Dromicodryas bernieri*	2				
	*Ithycyphus oursi*	1	1 (100%)	0.04		
	*Leioheterodon modestus*	1				
	*Madagascarophis colubrinus*	7	2 (29%)	0.2 ± 0.03 (0.17 − 0.23)		
	*Mimophis mahfalensis*	1				
	*Thamnosophis lateralis*	3				
	*Typhlops arenarius*	1				
		17	3 (18%)			
		73	5 (7%)		13 (18%)	

### Microscopic examination

**Figure 1.**
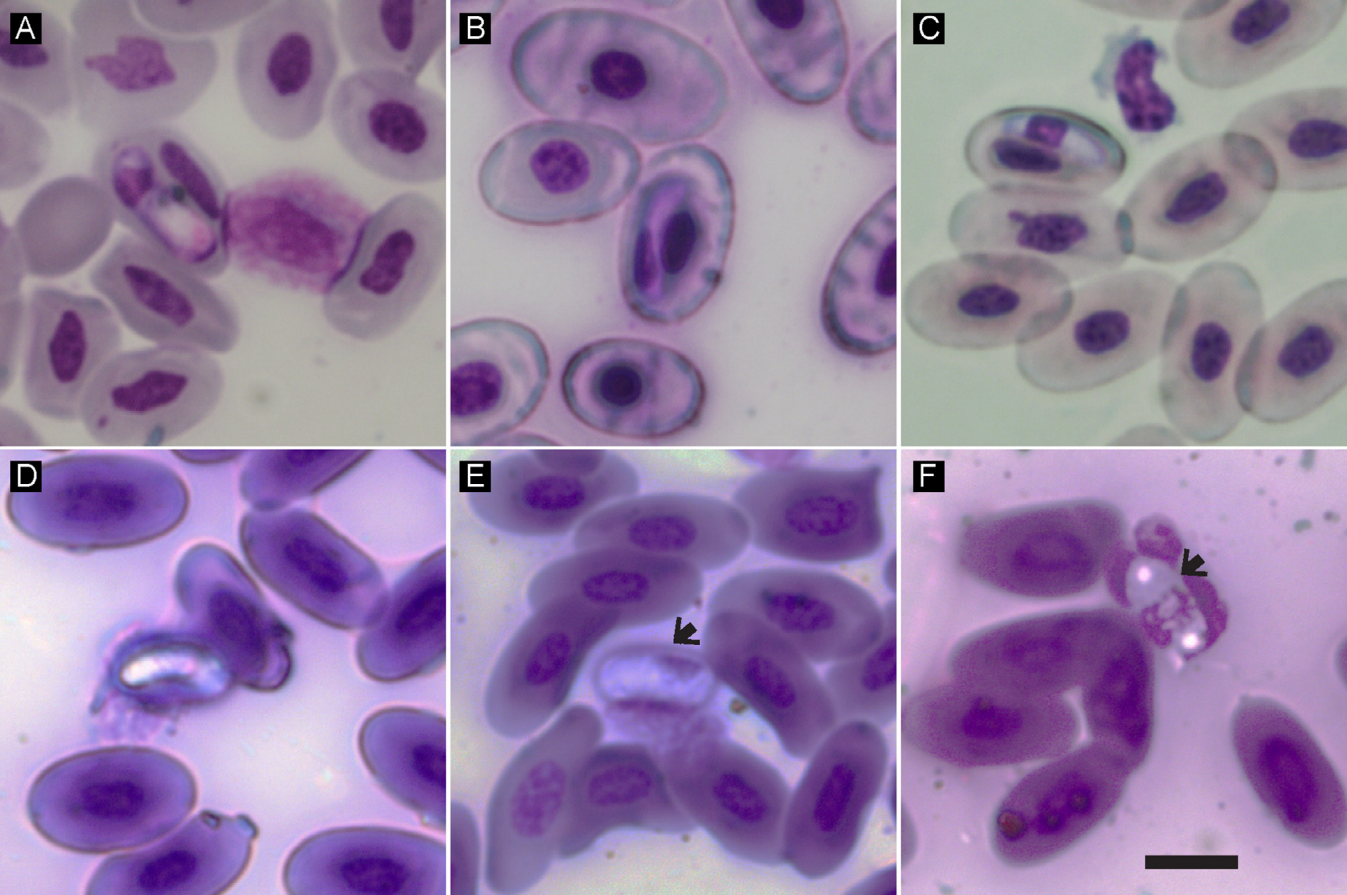
Hemogregarine mature gamonts in two snake species and one lizard species endemic to Madagascar. (A) *Hepatozoon* in *Ithycyphus oursi* (ACZC1932); (B) *Hepatozoon* in *Madagascarophis colubrinus* (ACZC1827); (C) *Hepatozoon* in *M. colubrinus* (ACZC1963); (D, E, F) hemogregarine infections in *Oplurus* sp. (×49). (F) Could represent a young stage based on the characteristics of the nucleus. *Hepatozoon* infecting *Furcifer* sp. is presented in Figure 2A. Arrows indicate hemogregarine parasites apparently inside leukocytes. Scale bar = 10 μm.

**Figure 2.**
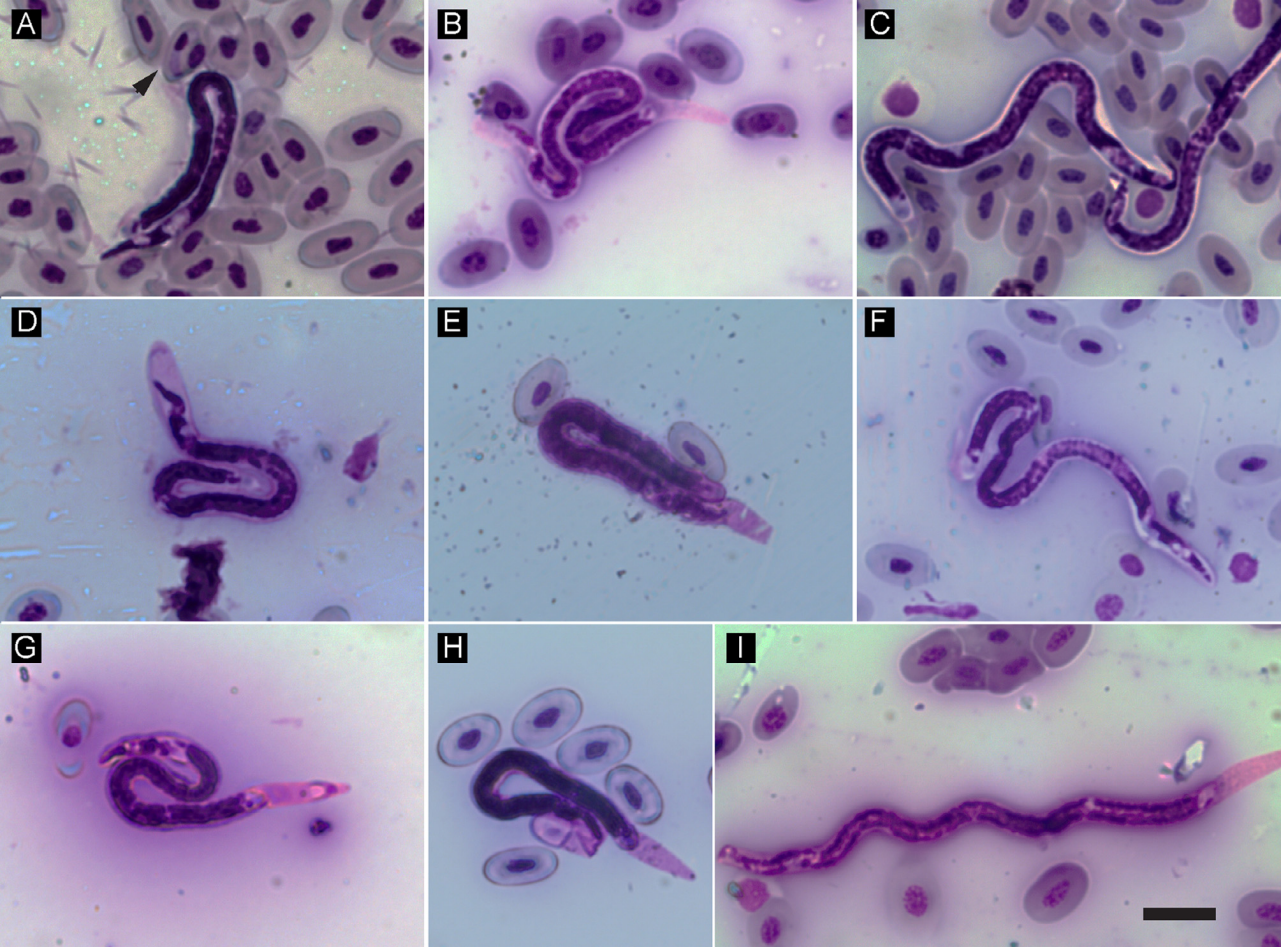
*Foleyella furcata* nematode infections in Malagasy chameleons of the genus *Furcifer*. (A) Mixed infection of *Hepatozoon* (arrowhead) and microfilariae in *Furcifer* sp.; (B) infection in a *F. lateralis* individual; (C, D) infections in two *F. oustaleti* individuals; (E, F, G) infections in three *F. verrucosus* individuals; (H, I) infections in two *Furcifer* sp. individuals. Scale bar = 20 μm.

Blood smears were screened initially at 400× magnification to search for extracellular hemoparasites, such as microfilariae, and at 1000× for intracellular parasites, using an Olympus CX41 microscope with an in-built digital camera (SC30) (Olympus, Hamburg, Germany). Prevalence was estimated as the proportion of infected hosts, and intensity of infection was estimated as the number of parasites per 5000 erythrocytes [13, 51] (Table 1). To reduce errors in manual counts, intensity counts for blood smears of infected individuals were counted three times and averaged. Mature hemogregarine gamonts and sheathed microfilariae in reptiles from Madagascar were observed (Figs. 1 and 2, respectively). Hemogregarine gametocytes and infected host erythrocytes were measured at 1000× magnification (see Table 3) and microfilariae at 400× magnification (see Table 4) using cell ^B software (basic image acquisition and archiving software; Olympus, Münster, Germany). For sheathed microfilariae, measurements include the sheathed part of the parasite. Length and width were taken using the horizontal and vertical distance tool for hemogregarines and polygon length tool for microfilariae, while the area and perimeter were taken using the area/perimeter tool in the Measure menu of the cell ^B software.

### DNA extraction, amplification, and sequencing

DNA was extracted from tissue using standard high-salt methods [50, 70]. Presence of hemogregarines was determined using PCR reactions with the apicomplexan primers HepF300 and HepR900 [83] targeting the overlapping part of the hemogregarine *18S* rRNA region. For positive samples we used the primers HEMO1 and HEMO2 [63] to amplify a partially overlapping fragment of the *18S* to obtain a longer portion of this gene. PCR cycling for the Hep primers consisted of 94 °C for 30 s, 60 °C for 30 s, and 72 °C for 1 min (35 cycles), while for HEMO primers the annealing temperature was 48 °C [27]. Microfilariae were detected in blood smears, and three pairs of primers were used to taxonomically identify these parasites: the *18S* rRNA gene [62], *COX1* and *12S* rRNA gene [40]. Negative and positive controls were run with each reaction. The positive PCR products were purified and sequenced by two commercial sequencing facilities (Macrogen Inc., Seoul, Korea; and CTM, Porto, Portugal). All sequences were performed in both directions. Sequences were deposited in GenBank under the accession numbers KM234619–KM234629 (*Foleyella furcata COX1* sequences), KM234630–KM234637 (*F. furcata 12S* rRNA gene sequences), KM234638–KM234645 (*F. furcata 18S* rRNA gene sequences), KM234646–KM234650 (hemogregarine *18S* rRNA gene sequences), and KM234651 (*Sarcocystis* sp. *18S* rRNA gene sequence).

### Molecular identification

The apicomplexan Hep primers amplified 4 of the 5 hemogregarine infections observed under the microscope. These were compared with data in GenBank using BLAST [4]. One sequence (sample ACZC1827 from *Madagascarophis colubrinus*, which could correspond to the gamonts observed under the microscope) displayed 4 heterozygous positions for the *18S* rRNA gene. Two haplotypes (KM234646 and KM234647) were derived from this sequence and included in the phylogenetic analysis. The HEMO set of primers only amplified 3 of the 5 hemogregarine infections and no mixed infections. For this reason, and since this produces similar tree topologies to those estimated using the longer fragment, we conducted phylogenetic analysis using the shorter fragment to include all sequences [48], although the longer fragments were deposited in GenBank when available. The sample infected with *Sarcocystis* sp. (KM234651 (sample ACZC1899) from *Oplurus* sp.) was identical to a published sequence (KC696571), thus no phylogenetic analysis was conducted for this parasite. Three genes were amplified for filarial nematodes, the *18S* rRNA gene, the *COX1* and *12S* rRNA gene. All *18S* and *12S* sequences were identical for the 8 samples analyzed, while for *COX1* four closely similar haplotypes were obtained. The BLAST results for the *12S* rRNA gene sequences (418 bp) indicated 99% similarity with the sequence AJ544841 from *F. furcata* and 93% identity with FR827906 from *Foleyella candezei* (Fraipont, 1882) [23] in GenBank. For this reason, we only present the results of the phylogenetic analyses of *COX1*. Sequences were analyzed using Geneious 6.0.3 [20], each electropherogram was carefully checked and aligned with MUSCLE algorithm implemented in this software. The new sequences were aligned with sequences retrieved from GenBank from various host species and the final datasets contained: 66 sequences of 590 bp in length for the *18S* rRNA gene fragment of hemogregarines; and 105 sequences of 590 bp for the *COX1* gene of filarial nematodes.

Two different phylogenetic analyses (Maximum Likelihood, ML, and Bayesian Inference, BI) were conducted for each group. ML analysis with random sequence addition (100 replicate heuristic searches) was used to assess evolutionary relationships, using the software PhyML 3.0 [26]. Support for nodes was estimated using the bootstrap technique [22] with 1000 replicates. The AIC criterion conducted in jModeltest 0.1.1 [64] was used to choose the best model of evolution and the parameters employed (TVM+G for hemogregarines and TIM3+I+G for filariae). BI was implemented using Mr. Bayes v.3.1 [32] with parameters estimated as part of the analysis. The analysis was run for 10 × 10^6^ generations, saving one tree each 1000 generations. The log-likelihood values of the sample points were plotted against the generation time and all the trees prior to reaching stationarity were discarded, ensuring that burn-in samples were not retained. Remaining trees were combined in a 50% majority consensus tree [32]. For hemogregarines, *Dactylosoma ranarum* (Lankester, 1882) [39] (HQ224958) and *Haemogregarina balli* Paterson and Desser, 1976 [60] (HQ224959) were used as outgroups, while for spirurid nematodes, *Ascaris lumbricoides* Linnaeus, 1758 [43] (JN801161), *Contracaecum rudolphii* Hartwich, 1964 [30] (NC014870), and *Heterakis isolonche* Linstow, 1906 [45] (FJ009626) were used as outgroups [40].

## Results

**Figure 3.**
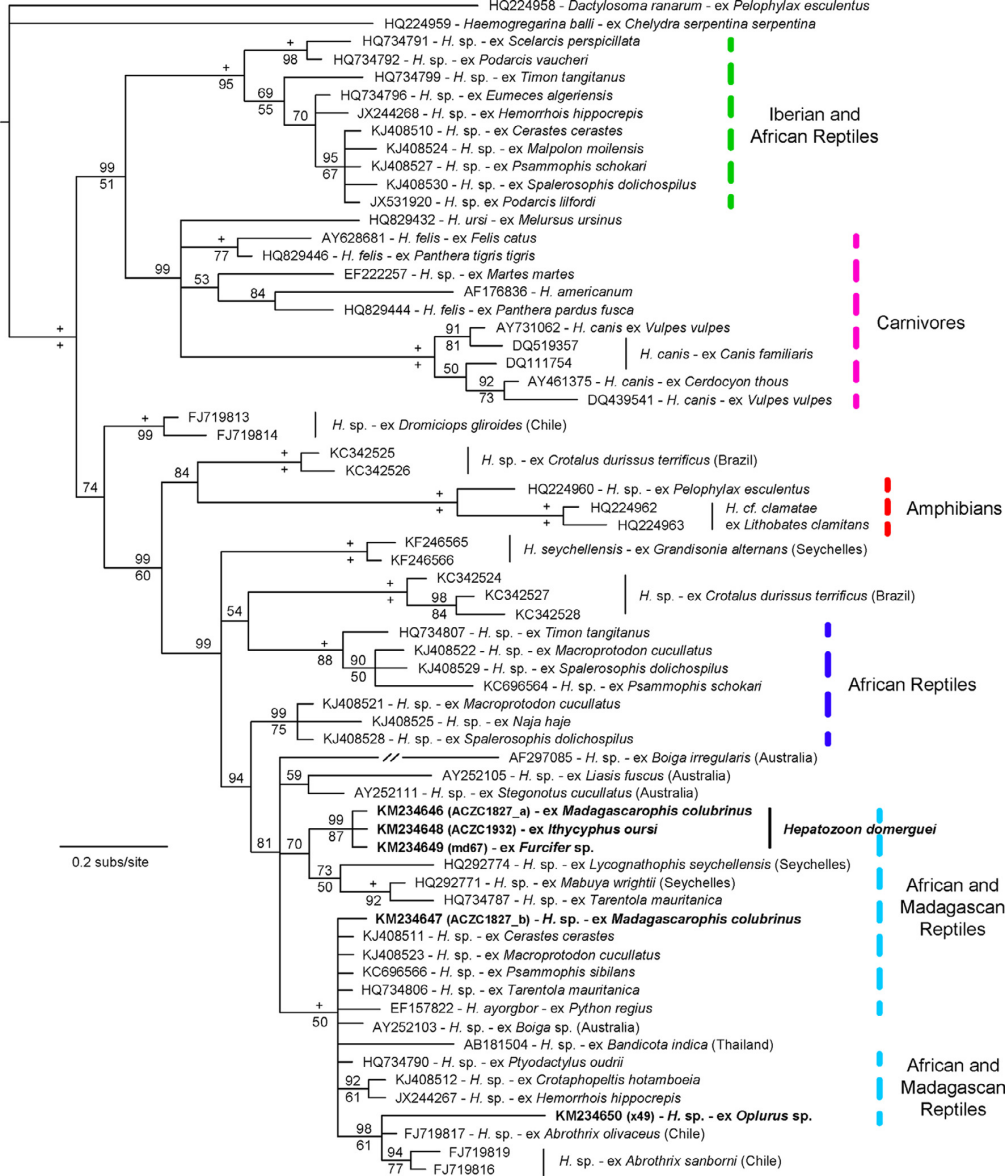
Tree derived from a Bayesian Inference analysis of the hemogregarine *18S* rRNA gene sequences. Bayesian Posterior Probability values are given above relevant nodes, and Bootstrap values for ML analyses below them. + indicates when support is 100. The branch for sequence AF297085 was shortened by 50%. The new sequences are in bold.

A total of 5 animals from different host species were infected with hemogregarines based on microscopy, resulting in an overall prevalence of only 7% (5/73) (Table 1). One chameleon was observed with both hemogregarine and filarial infections (Fig. 2A). Intensity levels were low overall, with *Ithycyphus oursi* Domergue, 1986 and *Oplurus* sp. having the lowest estimates, while the genus *Furcifer* had the highest (Table 1). Of the 5 hemogregarines identified by microscopy, 4 were sequenced for the *18S* rRNA gene and resulted in 3 haplotypes with some genetic divergence (Table 2 and Fig. 3). One haplotype was found in the predator-prey system composed of the chameleon *Furcifer* sp. (KM234649), and the snakes *I. oursi* (KM234648) and *Madagascarophis colubrinus* (KM234646). The mean measurements of gamonts from these genetically identical haplotypes (Table 3) match the descriptions of *Hepatozoon domerguei* from *M. colubrinus* (mean of 14 μm in length and 3 μm in width [38]). The second haplotype was found in the same infected individual of *M. colubrinus* (KM234647) and was more similar to other *Hepatozoon* sp. from continental African snakes (e.g. KJ508511) and lizards (e.g. HQ734806) (Fig. 3). Finally, the third haplotype was found in the iguanid lizard *Oplurus* sp. and clusters in a group with parasites identified from Chilean rodents (e.g. FJ719817). In fact, this parasite displayed distinct morphological characteristics (Figs. 1D, 1E, and Table 3), with some gamonts apparently inside leukocytes (Figs. 1E, 1F). The *Sarcocystis* sequence detected in the lizard *Tracheloptychus petersi* Grandidier, 1869 was identical (100%, 543 bp) to that reported in the snake *Psammophis schokari* Forsskål, 1775 (KC696571) from Algeria, and less similar to *Sarcocystis* spp. from lizards (AY015112 *Sarcocystis gallotiae* Matuschka and Mehlhorn, 1984 [52] from *Gallotia galloti eisentrauti* Bischoff, 1982 from Canary Islands, AY015113 *Sarcocystis lacertae* (Babudieri, 1932) [5] from *Podarcis muralis* (Laurenti, 1768) from Slovakia, and JQ762307 *Sarcocystis* sp. from *Podarcis lilfordi* (Günther, 1874) from the Balearic Islands).

**Table 2.** Estimates of evolutionary divergence between the hemogregarine three haplotypes obtained in this study. The number of base substitutions per site between sequences are shown. Analyses were conducted using the Maximum Composite Likelihood model [77]. There were a total of 565 positions in the final dataset. Evolutionary analyses were conducted in MEGA6 [78]. Haplotype 1 (Hap 1) is composed of sequences KM234646, KM234648, and KM234649; haplotype 2 (Hap 2) of sequence KM234647; and haplotype 3 (Hap 3) of sequence KM234650 (Fig. 3).

	Hap 1	Hap 2	Hap 3
Hap 1	–	–	–
Hap 2	0.013	–	–
Hap 3	0.035	0.025	–

**Table 3.** Microscopy measurements of hemogregarine intracellular parasites and infected host erythrocytes under 1000× magnification. An extracellular gametocyte was also detected for the sample ACZC1827 but was not included in these measurements (19.22 µm in length and 2.26 µm in width, similar to mean measurements for free gamonts in previous studies [38]). *n* refers to the number of hemogregarine gamonts or infected host cells measured per sample.

Host species	Code	Hemogregarines – Mean ± *SD* (min − max)					Host cell – Mean ± *SD* (min − max)				
		*n*	Vertical	Horizontal	Area	Perimeter	*n*	Vertical	Horizontal	Area	Perimeter
*Furcifer* sp.	md67	10	12.89 ± 0.94 (11.09 − 14.14)	4.31 ± 0.71 (2.95 − 5.10)	49.67 ± 5.36 (39.19 − 56.40)	34.78 ± 2.00 (31.23 − 38.36)	10	17.84 ± 1.45 (15.19 − 19.89)	11.02 ± 0.87 (9.97 − 13.01)	157.67 ± 11.48 (137.15 − 173.51)	51.74 ± 2.00 (48.77 − 54.79)
*Ithycyphus oursi*	ACZC1932	5	14.49 ± 0.46 (13.95 − 15.28)	3.77 ± 0.86 (2.56 − 5.21)	58.64 ± 9.21 (45.28 − 72.23)	39.12 ± 1.07 (37.59 − 40.66)	5	18.29 ± 1.35 (16.32 − 19.58)	10.82 ± 1.53 (8.13 − 12.76)	163.41 ± 31.87 (112.91 − 208.07)	52.84 ± 4.98 (45.46 − 58.57)
*Madagascarophis colubrinus*	ACZC1827	10	13.82 ± 0.56 (12.55 − 14.61)	2.97 ± 0.55 (1.83 − 3.59)	42.77 ± 8.73 (24.78 − 57.26)	36.43 ± 2.13 (32.60 − 40.81)	8	19.93 ± 1.59 (16.32 − 22.08)	11.20 ± 1.01 (9.97 − 13.20)	180.91 ± 10.63 (164.88 − 194.65)	56.80 ± 1.23 (55.10 − 58.87)
			13.73 ± 0.66 (11.09 − 15.28)	3.68 ± 0.71 (1.83 − 5.21)	50.36 ± 7.77 (24.78 − 72.23)	36.78 ± 1.73 (31.23 − 40.81)					
*Madagascarophis colubrinus*	ACZC1963*	10	11.53 ± 1.16 (10.12 − 14.40)	3.42 ± 0.35 (2.94 − 4.02)	35.93 ± 4.07 (30.85 − 42.80)	30.76 ± 2.33 (27.95 − 36.59)	9	15.99 ± 2.20 (12.54 − 19.30)	9.72 ± 1.41 (8.24 − 12.76)	123.34 ± 13.97 (92.00 − 142.14)	46.35 ± 3.42 (40.00 − 52.88)
*Oplorus* sp.	×49**	10	12.13 ± 0.51 (11.25 − 13.19)	5.88 ± 0.67 (4.72 − 7.01)	59.71 ± 5.90 (48.30 − 66.92)	34.26 ± 1.24 (32.38 − 36.08)	6	14.61 ± 1.21 (12.33 − 15.80)	10.54 ± 1.19 (8.24 − 11.84)	124.23 ± 15.90 (96.91 − 141.63)	45.15 ± 2.78 (41.68 − 49.10)

* PCR was negative for sample ACZC 1963.

** Sample ×49 presented morphologically distinct parasites (see Fig. 1), some of which apparently found inside leukocytes and yielded a genetically distinct *18S* rRNA gene haplotype (Fig. 3). Samples md67, ACZC1932 and ACZC1827 yielded identical *18S* rRNA gene sequences (Fig. 3), so a mean is presented for these as they may correspond to the same *Hepatozoon* species (*Hepatozoon domerguei*, see Discussion and Fig. 3). GenBank accession numbers are given in Figure 3.

**Figure 4.**
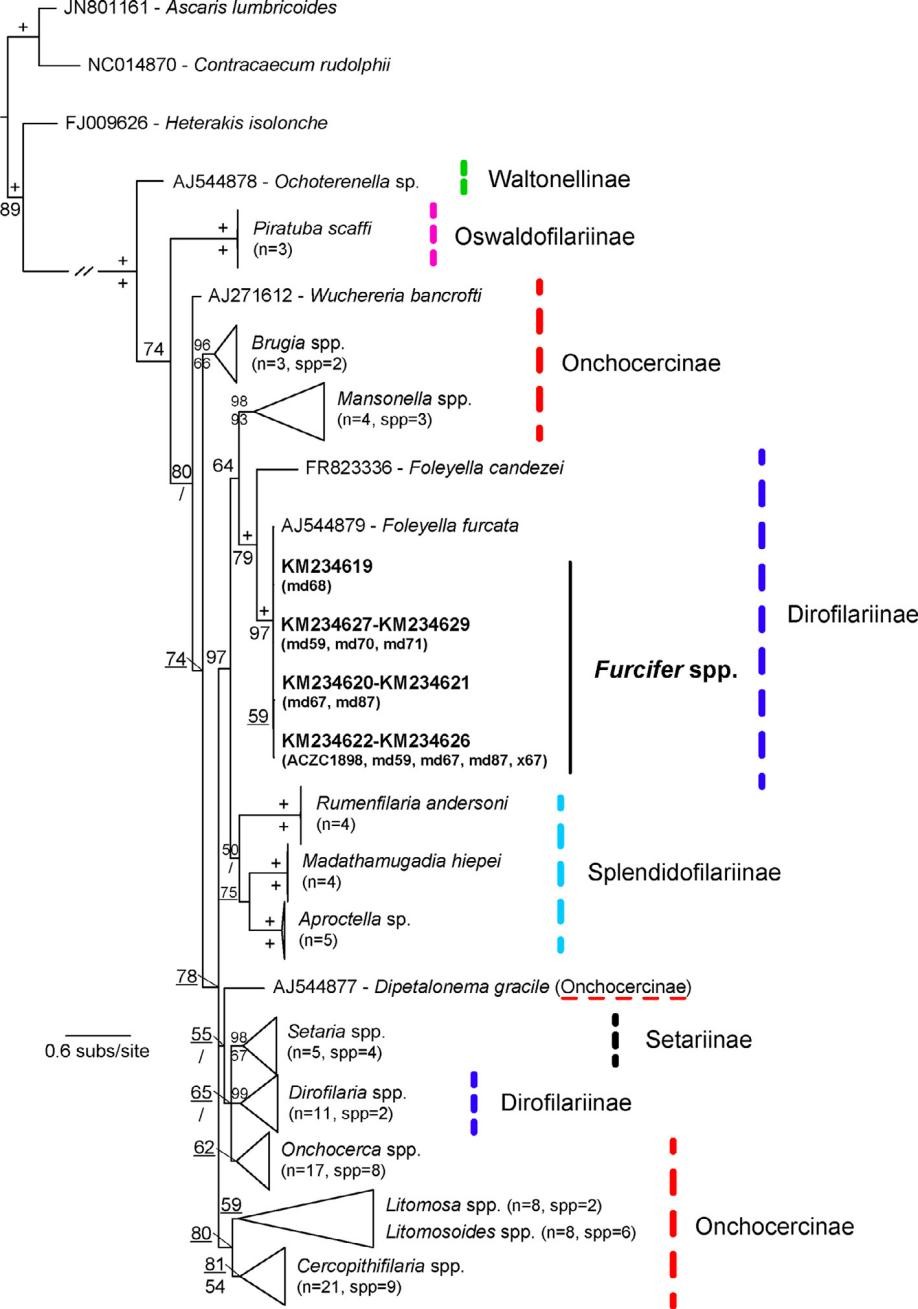
Tree derived from a Bayesian Inference analysis of the nematode *COX1* gene sequences. Bayesian Posterior Probability values are given above relevant nodes, and Bootstrap supports for ML analyses below them. The symbol + indicates when support is 100 and / when Maximum Likelihood topology differs. n refers to the number of sequences and spp. refers to the number of species that form the collapsed clade. The new sequences are in bold.

**Figure 5.**
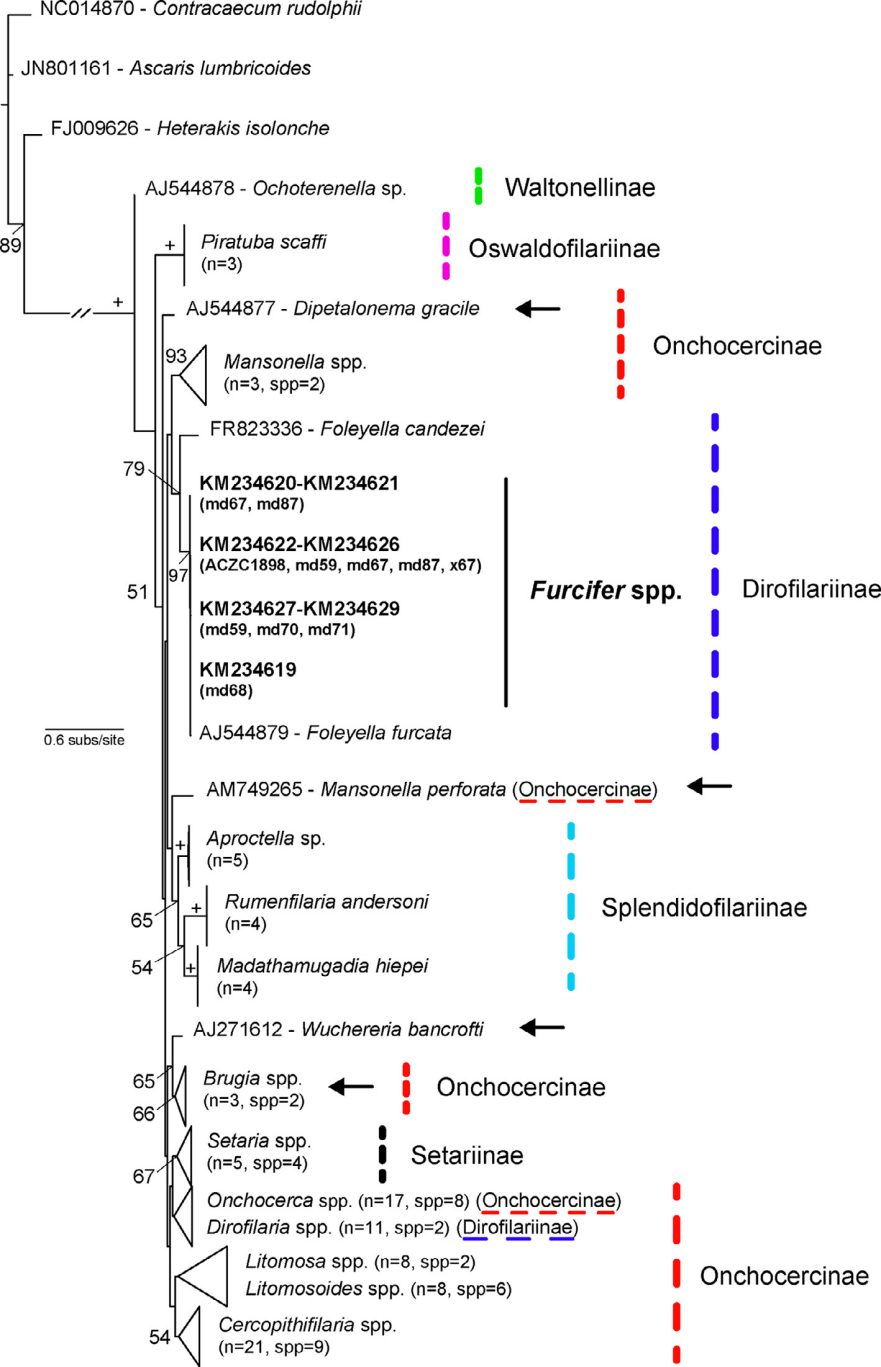
Tree derived from a Maximum Likelihood (ML) analysis of the nematode *COX1* gene sequences. The symbol + indicates when support is 100. *n* refers to the number of sequences and spp. refers to the number of species that form the collapsed clade. The new sequences are in bold and arrows indicate differences between the BI and ML phylogenetic analyses.

A relatively high number of chameleons from the genus *Furcifer* were infected with sheathed microfilariae (13/45, 37%) (Fig. 2) with varying infection intensities (Table 1). Mean microfilaria measurements per host species (Table 4) match the descriptions of *Foleyella furcata* in *Furcifer verrucosus* (range 125–157 μm and 6–7 μm) and are different from *Foleyella candezei* (83–96 μm and 6.5–7 μm [8]) and *Foleyella brevicauda* (225 μm and 7 μm [6]). *Foleyella* species are characterized by having a loose prominent sheath that completely encloses the body [10], as was observed in this study (Fig. 2). We sequenced 8 samples for three filarial genes (*18S* rRNA gene, *COX1* and *12S* rRNA gene). Sequences were identical for the *18S* and *12S* rRNA genes and the latter was similar to the previously published *F. furcata* (AJ544879; 99% identity). This is the first *18S* rRNA gene sequence for *F. furcata*, and the closest matches were *Loa loa* (Guyot, 1778) (DQ094173), *Onchocerca cervicalis* (Railliet and Henry, 1910) [67] (DQ094174), *Breinlia mundayi* (Spratt and Varughese, 1975) [76] (JF934735), *Dipetalonema* sp. (DQ531723), and *Setaria digitata* (Linstow, 1906) [45] (DQ094175) with 99% identity (895 bp). Phylogenetic analysis of the *COX1* gene confirms that these sequences belong to the species *F. furcata* (Figs. 4 and 5) and the tree topology resembles that of previous studies [40].

**Table 4.** Microscopy measurements of *Foleyella furcata* microfilaria in Giemsa-stained blood smears under 400× magnification (as confirmed by PCR sequencing, see Fig. 3). *n* refers to the number of microfilaria measured per sample.

Host species	Code	*n*	*Foleyella furcata* – Mean ± *SD* (min − max)			
			Length	Width	Area	Perimeter
*Furcifer lateralis*	×43*	10	148.46 ± 10.83 (131.06 − 168.77)	6.93 ± 0.43 (5.92 − 7.59)	969.32 ± 101.36 (766.98 − 1106.76)	332.43 ± 23.37 (293.12 − 376.73)
*Furcifer oustaleti*	md57*	10	155.88 ± 6.05 (144.31 − 168.24)	7.40 ± 0.47 (6.56 − 8.16)	1058.15 ± 52.58 (988.72 − 1156.45)	345.32 ± 15.85 (313.75 − 373.17)
	md59	10	125.84 ± 11.92 (106.80 − 145.62)	5.95 ± 0.37 (5.12 − 6.70)	682.61 ± 98.77 (543.62 − 819.30)	288.45 ± 27.16 (248.42 − 329.20)
			140.86 ± 8.99 (106.80 − 168.24)	6.68 ± 0.42 (5.12 − 8.16)	870.00 ± 75.68 (543.62 − 1156.45)	316.89 ± 21.52 (248.42 − 373.17)
*Furcifer verrucosus*	ACZC1898	10	118.75 ± 9.06 (105.31 − 135.48)	5.51 ± 0.46 (4.64 − 6.40)	608.21 ± 76.41 (478.54 − 714.50)	264.17 ± 20.84 (235.38 − 299.71)
	md56*	10	136.50 ± 17.88 (106.96 − 172.86)	6.34 ± 0.49 (5.76 − 7.24)	753.88 ± 154.45 (509.39 − 1076.02)	316.10 ± 49.67 (240.59 − 383.25)
	×67	10	122.02 ± 3.92 (116.16 − 128.87)	6.00 ± 0.51 (5.12 − 7.04)	663.08 ± 58.64 (587.52 − 805.07)	276.46 ± 9.06 (258.61 − 286.48)
			125.75 ± 10.29 (105.31 − 172.86)	5.95 ± 0.49 (4.64 − 7.24)	675.06 ± 96.50 (478.54 − 1076.02)	282.06 ± 22.84 (235.38 − 383.25)
*Furcifer* sp.	md67	10	115.84 ± 13.01 (97.73 − 137.74)	5.40 ± 0.76 (4.00 − 6.56)	524.46 ± 83.49 (408.76 − 639.08)	267.21 ± 32.22 (219.58 − 319.37)
	md68*	5	136.38 ± 26.72 (102.02 − 181.86)	6.18 ± 1.04 (4.16 − 7.04)	749.68 ± 195.57 (421.09 − 972.21)	303.14 ± 59.61 (231.40 − 407.32)
	md70**	10	108.51 ± 6.15 (100.14 − 117.74)	4.34 ± 0.29 (4.00 − 4.80)	396.60 ± 36.37 (350.00 − 480.38)	245.40 ± 13.00 (225.58 − 263.99)
	md71*	4	128.03 ± 6.83 (117.83 − 137.06)	6.24 ± 0.30 (5.76 − 6.58)	762.91 ± 73.76 (689.18 − 878.59)	281.34 ± 16.62 (256.14 − 302.82)
	md87**	10	117.17 ± 7.93 (100.81 − 130.29)	5.70 ± 0.64 (4.22 − 6.40)	624.74 ± 59.61 (525.00 − 713.83)	264.65 ± 17.43 (229.24 − 294.14)
	×46*	10	126.20 ± 7.80 (113.58 − 139.09)	6.23 ± 0.46 (5.44 − 6.88)	713.36 ± 55.59 (610.00 − 795.57)	283.11 ± 17.66 (257.93 − 311.08)
	×47*	10	142.37 ± 12.93 (124.13 − 163.79)	5.96 ± 0.39 (5.50 − 6.56)	790.25 ± 102.58 (638.49 − 940.39)	317.31 ± 26.90 (278.56 − 360.18)
			124.93 ± 11.62 (100.14 − 181.86)	5.72 ± 0.55 (4.00 − 7.04)	651.71 ± 86.71 (350.00 − 972.21)	280.31 ± 26.21 (219.58 − 407.32)

* Some microfilaria displayed larger sheaths that were included in the measurements.

** Many microfilaria were found in a coiled position, which complicates measurements and may explain lower values compared to the others. GenBank accession numbers are given in Figures 4 and 5.

## Discussion

This study shows that multiple parasites can be found in endemic reptile species from Madagascar. We detected hemogregarines at an overall low prevalence and intensity of infection in two snake species (*Ithycyphus oursi* and *Madagascarophis colubrinus*), a chameleon (*Furcifer* sp.) and an iguanid lizard (*Oplurus* sp.), while filarial infections were relatively high in chameleons (*Furcifer* genus).

We found an identical *Hepatozoon 18S* rRNA gene haplotype in the prey-predator system composed of the snakes *I. oursi* and *M. colubrinus*, and their prey *Furcifer* sp. [15]. This mode of transmission has been increasingly detected by molecular tools in reptiles from continental Africa [80, 81] and in mammals [2, 3, 50], and it has already been described for *Hepatozoon domerguei*, a hemogregarine species whose type host is *M. colubrinus* and type locality is Madagascar [38, 79]. Transmission from prey to predator is possible by ingestion of infective cysts in prey hosts that become infective to a predator and this is a plausible explanation for why the same haplotype was found in these host species. Based on this and the fact that *H. domerguei* gamonts are similar to those found in our study, we propose that this *Hepatozoon* haplotype is from *Hepatozoon domerguei*. However, this needs to be verified by identifying the developmental stages in arthropod vectors. *Hepatozoon* parasites can be transmitted by a wide range of arthropod vectors, such as mites, ticks, and mosquitoes, but the diversity and distribution of competent vectors of these parasites in Madagascar is limited, although sporogony was obtained in the arthropods *Culex quinquefasciatus* and *Anopheles stephensi* (Liston, 1901) [38]. The fact that an individual of *M. colubrinus* was infected with two haplotypes may indicate this host species may have been infected with different hemogregarine species [12, 79], although we did not find major morphological differences in the gamonts from this single individual that could indicate the presence of distinct *Hepatozoon* species in the blood. This may indicate that the second haplotype is a latent infection in the form of tissue cysts that is not visible (or present) in the blood, which may also be a case of dead-end infections, meaning that the parasite does not develop in this host species and is not transmitted further [82]. In addition, the other *M. colubrinus* individual infected with hemogregarine parasites in blood smears (ACZC1963) could not be amplified using the primers employed in this study, but given the distinct morphological characteristics (Table 3) this may indicate the presence of another hemogregarine species in this host. Hemogregarine taxonomy is problematic, with evidence that the genus *Hepatozoon* may be paraphyletic [9, 36, 74]. Thus, it is possible that some of these haplotypes belong to different hemogregarine genera. Future studies need to assess the developmental stages of these parasites and the use of faster-evolving genes [41] might help in taxonomic identification of these parasites. It is also worth mentioning that the Hep primers performed better than the HEMO primers by amplifying a broader range of parasites, as observed in other studies [28, 58], allowing for a better assessment of the distribution and diversity of these parasites.

To our knowledge, this is the first report of *Sarcocystis* parasites in the Peters’ keeled cordylid lizard *Tracheloptychus petersi*; however, sporozoites of *Sarcocystis* species have been previously reported from reptiles in Madagascar [84]. This lizard species is listed as Vulnerable under the IUCN Red List criteria and is a species with a decreasing population trend [68], thus it is important to assess the real prevalence of this parasite and investigate its implications for the host because *Sarcocystis* species are known to have adverse effects in some hosts [34]. This parasite is identical to that found in a snake from continental Africa, which provides further evidence that phylogenetic analysis of the *18S* rRNA gene of *Sarcocystis* does not reflect the relationships of their final hosts [81]. *Sarcocystis* parasites have a direct life cycle and are transmitted from infected prey to their predator [21], as it has been observed in recent molecular assessments [28, 81]. Given that the haplotype is identical between a lizard and a snake, it is reasonable to assume that this indicates a lizard-snake life cycle, which is not uncommon [85]. However, the fact that the same lineage of parasite is found in North African snakes and lizards endemic to Madagascar is indicative of low host specificity.

In this work we report a relatively high incidence of microfilarial infections in the chameleon genus *Furcifer*. Although morphological identification of nematodes to the species level requires the use of adult forms, by combining morphological characters and genetic information we were able to identity these microfilariae to the species level. Within the genus *Foleyella*, 4 species are known to infect reptiles, of which *F. furcata* and *F. brevicauda* have been previously reported in chameleons from Madagascar [6, 12]. Since both *F. furcata* and *H. domerguei* are transmitted by the southern house mosquito *Culex quinquefasciatus*, it is possible that the mixed infected chameleons observed in this study were infected by this vector. Future studies should determine the distribution of this vector in natural populations in Madagascar. Both hemogregarines and filarial nematodes are often asymptomatic but, when present at high intensities and/or in the presence of other hemoparasites, they may be associated with health implications and thus their impact in these wild endemic hosts should be further investigated. Sampling sizes across host species were not uniform and may not represent prevalence estimates. Thus, for a more realistic distribution of these parasites larger sampling studies are needed, as well as future studies that consider the ecological characteristics of the different geographical locations analyzed, host susceptibility and abundance of competent vectors. Altogether this and future epidemiological data should be considered when designing and employing conservation measures.
